# Identification of an *Alu* element‐mediated deletion in the promoter region of *GNE* in siblings with GNE myopathy

**DOI:** 10.1002/mgg3.300

**Published:** 2017-06-14

**Authors:** Jennifer Garland, Joshi Stephen, Bradley Class, Angela Gruber, Carla Ciccone, Aaron Poliak, Christina P. Hayes, Vandana Singhal, Christina Slota, John Perreault, Ralitza Gavrilova, Joseph A. Shrader, Prashant Chittiboina, Galen Joe, John Heiss, William A. Gahl, Marjan Huizing, Nuria Carrillo, May Christine V. Malicdan

**Affiliations:** ^1^ Medical Genetics Branch National Human Genome Research Institute National Institutes of Health Bethesda MD USA; ^2^ Therapeutics for Rare and Neglected Diseases National Center for Advancing Translational Sciences National Institutes of Health Bethesda MD USA; ^3^ Prevention Genetics Marshfield WI USA; ^4^ Surgical Neurology Branch National Institute of Neurological Disorders and Stroke National Institutes of Health Bethesda MD USA; ^5^ Office of the Clinical Director National Institute of Child Health and Human Development National Institutes of Health Bethesda MD USA; ^6^ Clinical Genomics and Department of Neurology Mayo Clinic Rochester MN USA; ^7^ Department of Rehabilitation Medicine Clinical Center National Institutes of Health Bethesda MD USA; ^8^ NIH Undiagnosed Diseases Program Common Fund Office of the Director National Institutes of Health Bethesda MD USA; ^9^ Office of the Clinical Director National Human Genome Research Institute National Institutes of Health Bethesda MD USA

**Keywords:** Alu‐SINE repeat, array‐CGH, copy number variant, genomic rearrangement, GNE isoforms, GNE myopathy, precision medicine, sialic acid

## Abstract

**Background:**

GNE myopathy is a rare genetic disease characterized by progressive muscle atrophy and weakness. It is caused by biallelic mutations in the *GNE* gene that encodes for the bifunctional enzyme, uridine diphosphate (UDP)‐N‐acetylglucosamine (GlcNAc) 2‐epimerase/N‐acetylmannosamine (ManNAc) kinase. Typical characteristics of GNE myopathy include progressive myopathy, first involving anterior tibialis muscle and sparing the quadriceps, and rimmed vacuoles on muscle biopsy. Identifying biallelic mutations by sequencing of the *GNE* gene confirms the diagnosis of GNE myopathy. In a subset of patients, diagnostic confirmation is challenged by the identification of mutations in only one allele, suggesting mutations in deep intronic regions or regulatory regions.

**Methods:**

We performed targeted sequencing and copy number variant (CNV) analysis of *GNE* in two siblings who clinically presented with GNE myopathy. Further molecular and biochemical studies were done to characterize the effect of a previously uncharacterized *GNE* mutation.

**Results:**

We report two siblings of Indian descent with characteristic features of GNE myopathy, including progressive skeletal muscle weakness initially involving the anterior tibialis, and rimmed vacuoles on muscle biopsy, in which a heterozygous mutation, p.Val727Met, was identified in both affected siblings, but no other deleterious variants in either coding region or exon–intron boundaries of the gene. Subsequent insertion/deletion analysis identified a novel 11.3‐kb deletion (Chr9 [GRCh37]: g.36257583_36268910del) encompassing the *GNE* promoter region, with breakpoints residing in *Alu* repeats. Gene expression analysis revealed reduced *GNE* mRNA and protein levels, confirming decreased expression of the deleted allele harboring the deletion.

**Conclusions:**

We have identified *GNE* as one of the genes susceptible to *Alu*‐mediated recombination. Our findings suggest that the deletion may encompass the promoter or another region necessary for *GNE* expression. In patients with typical manifestations of GNE myopathy and a single GNE variant identified, copy number variant (CNV) analysis may be useful in arriving at the diagnosis.

## Introduction

GNE myopathy (MIM 605820) is an autosomal recessive muscle disease caused by biallelic mutations in the *GNE* gene (MIM 603824) that encodes for the bifunctional enzyme, uridine diphosphate (UDP)‐N‐acetylglucosamine (GlcNAc) 2‐epimerase/N‐acetylmannosamine (ManNAc) kinase (Eisenberg et al. 2001; Huizing et al. 2014). This enzyme catalyzes the first two committed steps in the N‐acetylneuraminic acid (Neu5Ac, sialic acid) biosynthetic pathway (Hinderlich et al. 1997; Keppler et al. 1999; Lucka et al. 1999). Sialic acids are negatively charged carbohydrate derivatives that are widely distributed across different tissues with distinctive roles in each cell type including cell proliferation, cell interaction, and immune defense (Varki and Varki 2007). Decreased sialic acid production in GNE myopathy is thought to cause hyposialylation of glycoproteins or glycolipids in muscle fibers (Keppler et al. 1999; Tajima et al. 2005; Malicdan et al. 2009; Huizing et al. 2014; Patzel et al. 2014).

The GNE enzymatic activities, localization, and gene expression are highly regulated (Lucka et al. 1999; Ghaderi et al. 2007; Reinke et al. 2009a). Eight human *GNE* (*hGNE*) isoforms are reported; *hGNE1* is the major, ubiquitously expressed isoform, while isoforms *hGNE2* to *hGNE8* are differentially expressed and may act as tissue‐specific regulators of sialylation (Reinke et al. 2009a,b; Yardeni et al. 2011). Total *hGNE* mRNA is highest in liver and placenta, while skeletal muscle has low expression (Lucka et al. 1999; Yardeni et al. 2011).

GNE myopathy usually presents in early adulthood and progresses over decades (Huizing et al. 2014; Mori‐Yoshimura et al. 2014; Nishino et al. 2015). The anterior tibialis is typically the first affected muscle, resulting in foot drop and tripping. The disease progresses to involve other muscles of the lower and upper extremities, with relative sparing the quadriceps. Eventually patients require a wheelchair and assistance with activities of daily living (Huizing et al. 2014; Mori‐Yoshimura et al. 2014). The prevalence of GNE myopathy is 1:500 among Persian Jews (Eisenberg et al. 2001) and estimated 6/1,000,000 in the general population (Celeste et al. 2014). More than 150 causative *GNE* mutations have been reported for GNE myopathy (Celeste et al. 2014).

In this study, we describe two siblings of Indian descent who are compound heterozygous for a well‐described missense mutation and a novel 11.3‐kb deletion in the *GNE* gene. We discuss the effect of the deletion on GNE protein and *hGNE* gene expression, including effects on the different *hGNE* splice variants.

## Subjects and Methods

### Subjects

Both patients were evaluated under NIH protocol 11‐HG‐0218, “A Natural History of Patients with GNE Myopathy” (ClinicalTrials.gov Identifier NCT01417533). Open muscle biopsies were obtained under protocol 15‐HG‐0068, “An Open Label Phase 2 Study of ManNAc in Subjects with GNE Myopathy” (ClinicalTrials.gov Identifier NCT02346461). Both protocols were approved by the National Human Genome Research Institute Institutional Review Board. Patients provided written informed consent. Evaluations were performed at the NIH Clinical Center and included medical and family history, as well as comprehensive physical, biochemical, and imaging studies. Muscle imaging was performed using a single 3‐T whole‐body MRI system (Verio, Siemens Medical Systems, Erlangen, Germany).

### DNA analysis

DNA was isolated from whole blood. Mutation analysis of the *GNE* coding exons (NM_001128227.2) was performed by Sanger sequencing (primer sequences available upon request). CNV analysis was performed on Prevention Genetics’ high‐density gene‐centric comparative genomic hybridization array (aCGH); a custom‐designed oligonucleotide array (720K probes; Oxford Gene Technology). Analysis was performed using Cytosure Interpretation Software (Oxford Gene Technology, Begbroke, UK). Primer sequences used for PCR confirmation and Sanger sequencing across the deletion are provided in Table S1.

### RNA expression analysis

Total RNA was isolated from whole blood (PAX gene Blood RNA Kit; Ambion, Inc., Austin, TX, USA). First strand cDNA was synthesized with high‐capacity RNA‐to‐cDNA kit (Applied Biosystems). Quantitative real‐time PCR (qPCR) was performed on 100 ng/μL cDNA using power SYBR Green mix (Applied Biosystems, Foster City, CA, USA) and Bio‐Rad qPCR machine with standard qPCR parameters to analyze *hGNE* isoform expression compared to *POLR2A*, using the comparative C_T_ method (Livak and Schmittgen 2001). For tissue‐specific expression studies, human multiple tissue cDNA panels (Clontech Laboratories, Mountain View, CA, USA) were amplified with isoform‐specific primers (primer sequences available upon request) and visualized on a 2% Agarose gel.

### Immunoblotting

Epstein–Barr virus (EBV) transformed lymphoblasts from patients were grown in RPMI medium. Cell lysates were subjected to immunoblotting, using a 4–20% Tris‐Glycine gel (Life Technologies, Carlsbad, CA, USA) and nitrocellulose membranes (Invitrogen, Carlsbad, CA, USA). Membranes were blocked with Li‐Cor blocking buffer (Li‐Cor Biosciences, Lincoln, NE, USA), and incubated with goat polyclonal anti‐GLCNE (GLCGNE [T‐19]; Santa Cruz Biotechnology, Santa Cruz, CA, USA) and mouse monoclonal anti‐ACTB (anti‐*β*‐actin; Sigma‐Aldrich, St. Louis, MO, USA) antibodies followed by incubation with secondary antibodies (Li‐Cor Biosciences). Protein bands were visualized and quantified using Odyssey^®^ imaging system (LI‐COR Biosciences).

## Results

**Figure 1:**
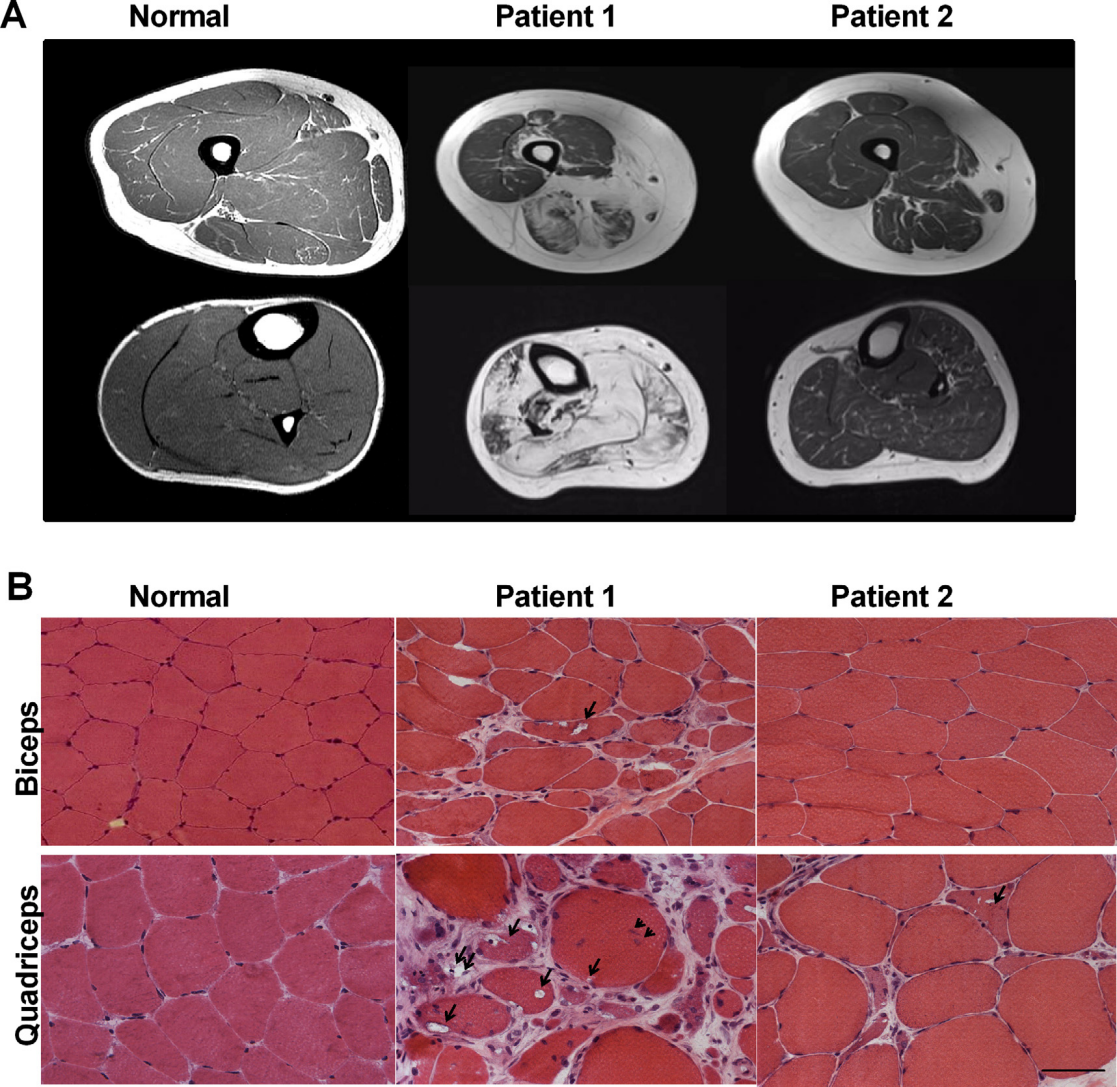
Muscle imaging and pathology. (A) T_1_‐weighted MRI of the thigh (*upper panel*) and lower leg (*lower panel*). Atrophy of hamstrings and lower leg muscles (Patient 1) and the proximal anterior tibialis (Patient 2) gave rise to fatty infiltration, apparent as white on the MRI. (B) Muscle biopsies of biceps brachii and lower extremity muscles (gastrocnemius medialis in Patient 1, anterior tibialis in Patient 2, quadriceps femoris in Normal) show characteristic findings of GNE myopathy, including rimmed vacuoles (*arrows*), fatty and fibrous tissue replacement (*double arrows*), marked variation in fiber size, and central nucleation (*arrowhead*). Note that the biceps muscle of Patient 2 appears normal, except for a mild variation in fiber size. Scale bar = 50 microns.

**Figure 2:**
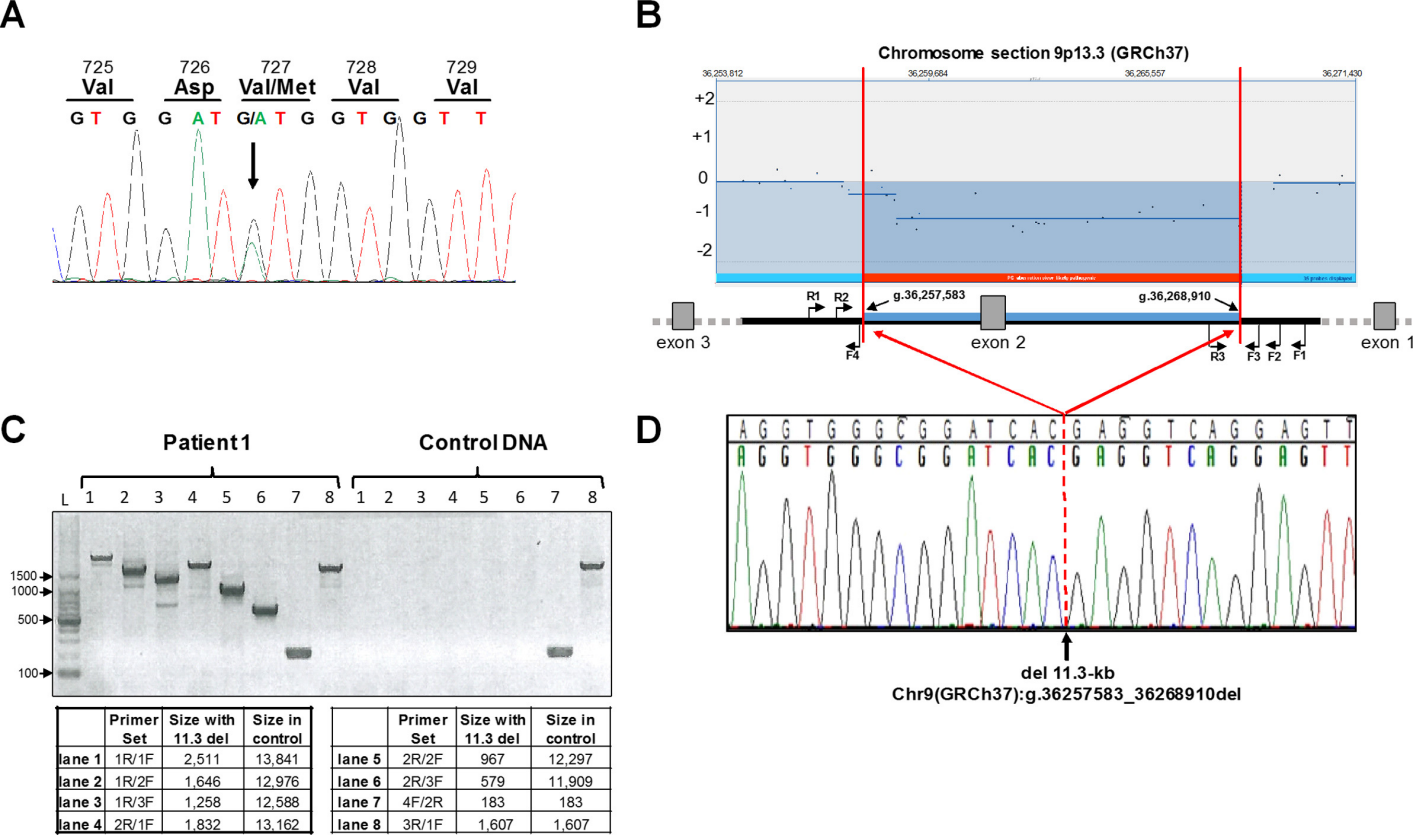
*GNE* mutation analysis. (A) Sanger sequencing of *GNE* exon 13 confirmed the heterozygous *GNE* mutation [NM_001128227.2:c.2179G>A;p.Val727Met] in both siblings (Patient 1 displayed). (B) Enlarged region of chromosome 9 (GRCh37) aCGH analysis that showed heterozygosity of a large (>10 kb) region in both patients. Primer locations for deletion analysis are indicated (not to scale, note that *GNE* gene is transcribed on reverse strand). (C) The size and breakpoints of the deletion were established by PCR analysis across the deletion; expected fragment sizes for each primer set are indicated. (D) Sanger sequencing across the deletion (Primer 1R; reverse sequence shown) determined the exact breakpoints and deletion size as: Chr9(GRCh37):g.36257583_36268910del (del 11,328‐bp).

Patients 1 and 2 are siblings from a nonconsanguineous family of Indian descent with no prior family history of neurological or muscle disease. Patient 1 is a female who presented at 25 years of age with ankle pain, tripping, and abnormal gait. When she was first evaluated at 27 years of age, she had bilateral foot drop, waddling gait, inability to stand on her toes or heels, or to stand up from a squat. Previous targeted sequencing for *MYH2* (MIM 160740) and *VCP* (MIM 601023) genes did not reveal pathogenic variants. When she was evaluated at the NIH at 28 years of age, the disease had progressed to involve lower and upper extremity muscles. The patient used bilateral ankle foot orthotics and a cane for ambulation, had difficulty walking, climbing upstairs, standing from a squat, opening jars, and combing her hair. She denied problems with diplopia, facial weakness, chewing, swallowing, or sensation. On physical examination, she was unable to stand on her toes or walk on her heels, and had steppage gait. Otherwise she had a normal cranial nerve examination, no facial weakness, and normal coordination, sensation, and deep tendon reflexes. There was no respiratory or cardiac involvement. Electrocardiogram, echocardiogram, and pulmonary function tests, including supine to sitting, were normal. Creatine kinase was 167 U/L. Muscle biopsy showed characteristic findings of GNE myopathy, including marked fiber size variation, rimmed vacuoles, and absence of inflammation in both biceps brachii and gastrocnemius muscles; in addition, the gastrocnemius muscle showed marked fibrosis (Fig. 1). Muscle strength was evaluated by Quantitative Muscle Strength Assessment (QMA; Aeverl Medical, Gainesville, GA, USA). The results are expressed as percent of predicted strength for each muscle accounting for age, gender, height, and weight (The National Isometric Muscle Strength (NIMS) Database Consortium 1996). Ankle dorsiflexion strength was <1% of predicted bilaterally and knee extension >50% of predicted, showing a characteristic pattern of GNE myopathy with advanced ankle dorsiflexion weakness and relative sparing of the quadriceps. T_1_‐weighted MRI showed atrophy of hamstrings and lower leg muscles (Fig. 1A). Sequencing of the *GNE* gene revealed a single disease‐causing mutation c.2179G>A (p.Val727Met) in exon 13 (Fig. 2A). Deletion/duplication analysis of the *GNE* gene revealed a novel 11,328‐bp deletion [c.51+7981_52‐8189del] (or Chr9(GRCh37):g.36257583_36268910del (Fig. 2B), not previously reported nor found in other genomic databases. Primer sets around the suspected deletion breakpoints amplified fragments across the deletion (Table S1, Fig. 2B,C). Sanger sequencing revealed the exact deletion breakpoints (Fig. 2D), consistent with the expected sizes of the PCR fragments (Fig. 2B).

Patient 2, the male brother of Patient 1, was diagnosed presymptomatically by genetic testing at the age of 24 years and harbors the same *GNE* gene variants as his affected sister. He was evaluated at 25 years of age at the NIH. At that time, he reported difficulty running and occasional ankle pain. On physical examination, he was unable to walk on his heels and walked with difficulty on his toes, but no other abnormalities could be identified. There were no cardiac or respiratory abnormalities identified by electrocardiogram, echocardiogram, or pulmonary function tests. Creatine kinase was 136 U/L. Tibialis anterior biopsy showed fiber size variation and scattered fibers with rimmed vacuoles; biceps muscles showed mild variation in fiber size (Fig. 1). Muscle strength by QMA showed mild weakness of different muscle groups and was significant for decreased ankle dorsiflexion strength of 25% of predicted bilaterally, T_1_‐weighted MRI showed atrophy of the proximal anterior tibialis (Fig. 1A), which confirmed the physical examination findings.

Because of the characteristic presentation of anterior tibialis weakness presenting in young adults, relative sparing of the quadriceps, family cosegregation with autosomal recessive inheritance (data not shown), and presence of one heterozygous disease‐associated *GNE* variant, we evaluated the pathogenicity of the 11,328‐bp deletion.

**Figure 3:**
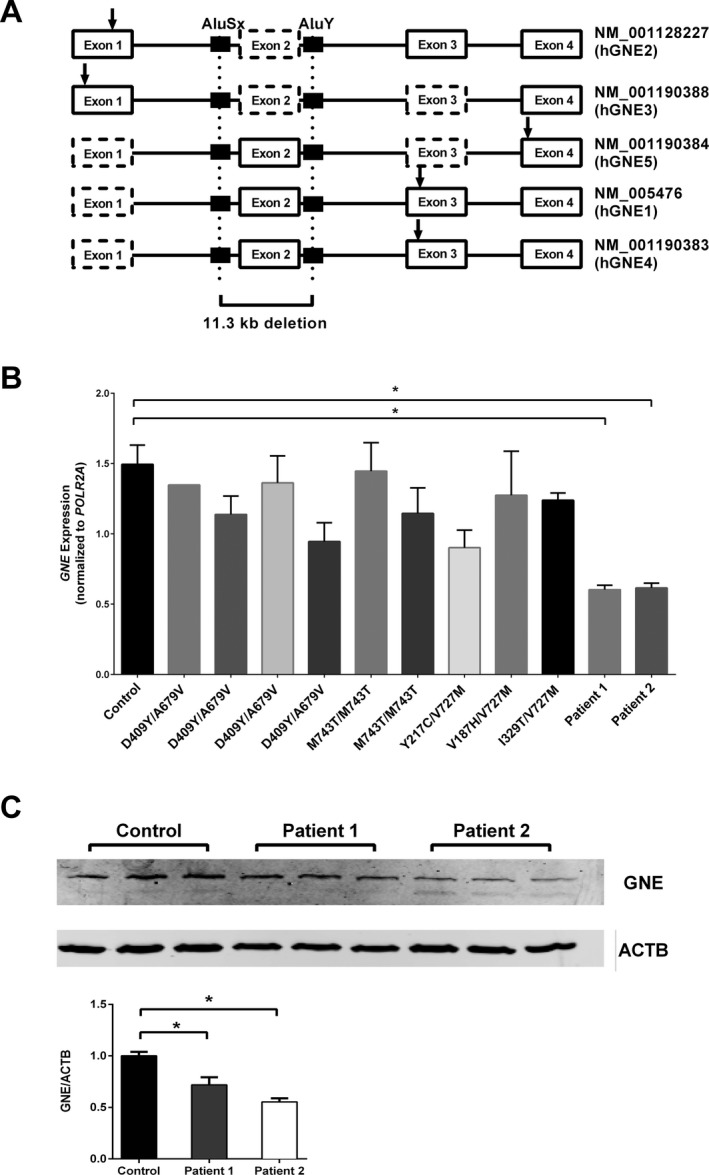
*GNE* gene and protein expression. (A) Display of the 5′ terminal region of the five *GNE* splice variants listed in GenBank. Exons represented by dotted boxes are absent in respective transcripts. The 11.3 kb deletion breakpoints (*vertical dotted lines*) within *Alu*‐repeat regions (black boxes) and the start codon (ATG) position (*arrow*) of each isoform is shown. The deletion is located deeply intronic for two isoforms (*hGNE2* and *hGNE3*) and in the 5′ UTR and promoter region of others (*hGNE1*,*hGNE4*, and *hGNE5*). (B) Blood *GNE* mRNA levels normalized with *POLR2A*. Patients 1 and 2, harboring the 11.3 kb *GNE* deletion in combination with a missense, have reduced *GNE* expression, as compared to other GNE myopathy patients whose biallelic missense mutations in *GNE* are mentioned. *P <* *0.05. (C) Measurement of GNE protein expression in lymphoblastoid cell lines from control and Patients 1 and 2. Expression levels were normalized with ACTB, and expressed as a ratio with control (lower bar graph). *P <* *0.05.

GenBank describes five of the eight human *GNE* splice variants (Yardeni et al. 2011): *hGNE1* (NM_005746), *hGNE2* (NM_001128227), *hGNE3* (NM_001190388), *hGNE4* (NM_001190383), and *hGNE5* (NM_001190384) (Fig. 3A). The 11.3 kb deleted area in our patients is located in the deep intronic (intron 1) region for two transcripts (*hGNE2* and *hGNE3*) and in the 5′ UTR and promoter region of three others (*hGNE1*,* hGNE4*, and *hGNE5*) (Fig. 3A). Online tools predicted a splicing defect (www.interactive-biosoftware.com/alamut-visual) due to the deep intronic 11.3 kb deletion in *hGNE2* and *hGNE3*. We measured expression of *hGNE2* and *hGNE3*, with transcript‐specific primers by PCR in whole blood (lymphoblast) cDNA, which showed no amplification products in controls (Fig. S1, EBV) nor in cDNA from both patients (not shown); this indicated that these *hGNE* transcripts are not expressed in blood lymphoblasts. PCR on multiple tissue cDNA panels showed expression of *hGNE2* in kidney, liver, and heart; and *hGNE3* in kidney, liver, and muscle (Fig. S1), similar to previous results (Yardeni et al. 2011), thereby validating our primer choice for these splice variants.

The 11.3 kb deletion most likely affects the three other *hGNE* splice variants (*hGNE1*,* hGNE4*, and *hGNE5*) by altering mRNA expression, stability, and/or translation. We designed a primer pair that specifically amplified these three transcripts together and measured *GNE* mRNA expression in blood by quantitative PCR. There was a 40–50% reduction in *hGNE* expression in both affected siblings (Fig. 3B), when compared to control and other patients with biallelic mutations in *GNE*. Immunoblotting showed a significant decrease (55–70%) in the amount of GNE protein expressed in lymphoblastoid cells of both patients compared to control (Fig. 3C), likely resulting from the decreased mRNA expression.

## Discussion

In this study, we describe two siblings of Indian descent with characteristic clinical and histopathological manifestations of GNE myopathy with compound heterozygous *GNE* mutations. One mutation is a missense variant, p.Val727Met in the GNE‐kinase domain, a common mutation in patients of Indian ancestry with GNE myopathy (Nalini et al. 2013; Celeste et al. 2014). The other mutation is a novel 11.3‐kb deletion located in the 5′ UTR and promoter region of *hGNE*. The site of the deletion is deeply intronic to be detected by conventional Sanger sequencing. Therefore, genomic rearrangement (copy number variant [CNV]) analysis should be considered when a single mutation is identified in patients with autosomal recessive inheritance and characteristic manifestations of GNE myopathy.

Indeed, while preparing this report, a study reported 9 novel CNVs in 13 Asian GNE myopathy patients with a previously identified monoallelic *GNE* mutation. The CNVs ranged from 0.5 kb to >40 kb, and most appeared to include *GNE* exon 2 and were flanked by *Alu* repeats. The study identified *GNE* as an *Alu*‐rich gene, and particularly exon 2 as a hotspot of genomic *GNE* rearrangements (Zhu et al. 2017). The 11.3 kb deletion in our siblings (annotated del g.13,132–24,459 on NG_008246.1 for direct comparison), was not identified in this previous study (nor were either of the breakpoints), but did include exon 2 and was indeed flanked by *Alu* repeats of the *SINE/Alu* family; *AluSx* on the 5′ end and *AluY* on the 3′ end of the deletion (Fig. 3A), which likely underlie the rearrangement/deletion mechanism. This novel 11.3 kb deletion should be checked in patients of Indian descent in which no or only one *GNE* variant was identified, such as the 4 Indian patients with monoallelic mutations reported by Nalini et al. (2013).

We demonstrated that the 11.3 kb deletion affects expression of isoforms *hGNE1*,* hGNE5*, and *hGNE4*, likely because of degradation due to nonsense‐mediated decay or aberrant expression due to promoter mutation of the deleted allele. The *hGNE1* transcript is the major GNE isoform, as it is expressed in most tissues tested and its protein contains the highest epimerase and kinase activity (Reinke et al. 2009b; Yardeni et al. 2011). The *hGNE1* transcript contains exon 2 in its noncoding 5′ UTR, and a deletion of this exon may explain decreased mRNA transcription in our patients (Fig. 3B).

Interestingly, while the *hGNE* mRNA expression is ~50% reduced in our patients’ lymphoblastoid cells, the amount of residual GNE protein in these cells is only moderately reduced (55–70% of normal) (Fig. 3C). This might be due to the cumulative translation of missense mutant alleles and/or expression of other unknown isoforms. Of note, biallelic GNE missense mutations in other patients with GNE myopathy appeared to not affect *GNE* mRNA expression (Fig. 3B), which was not previously reported. It is difficult to predict the effect of the patients’ 11.3 kb deletion and p.Val727Met substitution on GNE enzyme activity, since we could not completely assess the protein expression of all GNE isoforms due to lack of specific antibodies and/or appropriate patients’ tissue; an additional complication is the fact that GNE homodimerizes to oligomeric structures with different enzyme activities (Ghaderi et al. 2007; Reinke et al. 2009b).

The 11.3 kb deletion most likely spares the *hGNE2* and *hGNE3* isoforms, since these transcripts do not contain exon 2 (Fig. 3A). Therefore, it is possible that accumulation of substrates due to reduced *hGNE1, hGNE5*, and *hGNE4* expression promotes overexpression of *hGNE2* and *hGNE3* as a compensatory mechanism. Unfortunately, expression in tissues could not be evaluated due to limited patient tissue samples. *hGNE2* and *hGNE3* were not expressed in control blood lymphoblasts (EBV, Fig. S1). Both transcripts were expressed only in selected tissues including liver and kidney, consistent with earlier findings (Yardeni et al. 2011). We also detected *hGNE2* expression in heart and *hGNE3* in skeletal muscle (Fig. S1), in contrast with a previous report (Yardeni et al. 2011).

GNE myopathy is a slowly progressive muscle disease. There have been reports of variability in the disease, even among siblings (Ro et al. 2005). However, both patients had onset of symptoms at 25 years of age. Patient 1 is older than her brother, and therefore, at the time of evaluation, she was at a more advanced stage of progression than her brother. At follow‐up visits, both patients have shown a similar rate of progression.

In conclusion, this study emphasizes considering genomic rearrangement/deep intronic and CNV analysis in the *GNE* gene, with emphasis on the exon 2 containing region, in patients with characteristic features of GNE myopathy, in which no biallelic pathologic *GNE* variants could be identified. Such analysis could improve patient diagnosis, since biallelic disease‐causing mutations in the *GNE* gene ultimately confirm the diagnosis. A delayed or incomplete diagnosis not only causes emotional hardship for the patient but also delays proper management of the disease and may influence eligibility to enroll in clinical trials and/or response to therapy. Furthermore, our findings suggest that the area encompassed by the deletion harbors a region that plays a critical role in *GNE* expression that should be further investigated.

## Conflict of Interest

None declared.

## Supporting information


**Figure S1** (A) PCR amplification of tissue‐specific cDNA from selected human tissues (human multiple tissue cDNA panels, Clontech Laboratories) or EBV cells (Epstein–Barr virus [EBV] transformed human lymphoblasts) with primers specifically amplifying *hGNE2* (114 bp), *hGNE*3 (109 bp), or a C‐terminal common region expressed in all GNE transcripts (392 bp).

**Table S1** Primer sequences on chromosome 9p13.3 (GRCh37) used for Fig. 2B*.
