# Supplementary figures and images for: Identification of an Alu element‐mediated deletion in the promoter region of GNE in siblings with GNE myopathy
Source: Mol Genet Genomic Med. 2017 Jun 14;5(4):410–7. doi: 10.1002/mgg3.300 (PMC5511805; doi:10.1002/mgg3.300)

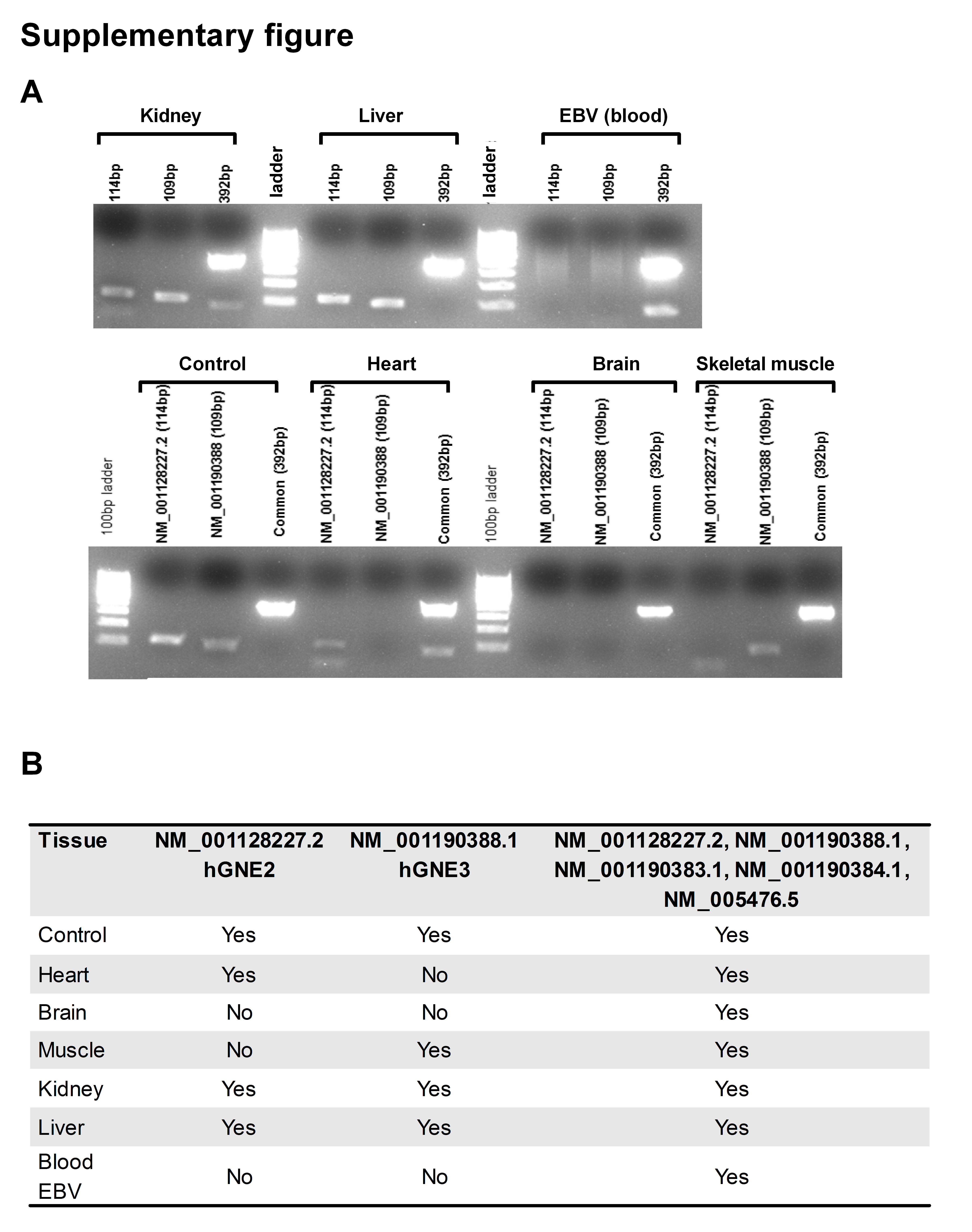

Supplement: Supplementary file 1 — Figure S1 (A) PCR amplification of tissue‐specific cDNA from selected human tissues (human multiple tissue cDNA panels, Clontech Laboratories) or EBV cells (Epstein–Barr virus [EBV] transformed human lymphoblasts) with primers specifically amplifying hGNE2 (114 bp), hGNE3 (109 bp), or a C‐terminal common region expressed in all GNE transcripts (392 bp). [file MGG3-5-410-s001.tif]
