# Supplementary material for: Identification of an Alu element‐mediated deletion in the promoter region of GNE in siblings with GNE myopathy
Source: Mol Genet Genomic Med. 2017 Jun 14;5(4):410–7. doi: 10.1002/mgg3.300 (PMC5511805; doi:10.1002/mgg3.300)
Supplement: Supplementary file 2 — Table S1 Primer sequences on chromosome 9p13.3 (GRCh37) used for Fig. 2B*. [file MGG3-5-410-s002.docx]

**Identification of an *Alu* element-mediated deletion in the promoter region of *GNE* in siblings with typical manifestations of GNE myopathy**

Jennifer Garland^1,2,*^, Joshi Stephen^1,*^, Bradley Class^2,*^, Angela Gruber^3^, Carla Ciccone^1^, Aaron Poliak^1^, Christina P. Hayes^4^, Vandana Singhal^2^, Christina Slota^2^, John Perreault^2,5^, Ralitza Gavrilova^6^, Joseph A. Shrader^7^, Prashant Chittiboina^4^, Galen Joe^7^, John Heiss^4^, William A. Gahl^1,8^, Marjan Huizing^1^, Nuria Carrillo^1,2,*^, May Christine V. Malicdan^1,8,*^

1. Medical Genetics Branch, National Human Genome Research Institute, National Institutes of Health, Bethesda, 20892 Maryland, USA
2. Therapeutics for Rare and Neglected Diseases, National Center for Advancing Translational Sciences, National Institutes of Health, Bethesda, 20892 Maryland, USA
3. Prevention Genetics, Marshfield, 54449 Wisconsin, USA
4. National Institute of Neurological Disorders and Stroke, National Institutes of Health, Bethesda, 20892 Maryland, USA
5. Office of the Clinical Director, National Institute of Child Health and Human Development, National Institutes of Health, Bethesda, 20892 Maryland, USA
6. Clinical Genomics and Department of Neurology, Mayo Clinic, Rochester, 55905, Minnesota, USA
7. Department of Rehabilitation Medicine, Clinical Center, National Institutes of Health, Bethesda, 20892 Maryland, USA
8. NIH Undiagnosed Diseases Program, Common Fund, Office of the Director, National Institutes of Health, Bethesda, 20892 Maryland, USA and the Office of the Clinical Director, National Human Genome Research Institute, National Institutes of Health, Bethesda, 20892 Maryland USA

**SUPPLEMENTARY TABLE S1**

**Primer sequences on chromosome 9p13.3 (GRCh37) used for Figure 2B***

| **Primer**  **Name** | **Primer Sequence** | **Primer location**  **(Chr 9, GRCh37)** |
| --- | --- | --- |
| **F1** | 5'-TTTGATGCCCGATTTTCTTG-3’ | 36,270,430 |
| **F2** | 5'-ATGACTCCATGATTTTGAGTCTGA-3’ | 36,269,565 |
| **F3** | 5'-AGAGAGAGGTTGAGGCCTTATG-3’ | 36,269,177 |
| **F4** | 5'-GAGGGGTGGGGTACAGTTTC-3’ | 36,257,451 |
| **R1** | 5'-CCGTCCATTATAAATGCTGACA-3’ | 36,256,590 |
| **R2** | 5'-AAATCGCCCTTTCATCAGTG-3’ | 36,257,269 |
| **R3** | 5'-GGAACTGAAGAATAGCCCACAG-3’ | 36,268,824 |

* Primer locations are indicated in Figure 2B. Note that Fig 2B image shows the reverse strand for the *GNE* gene.
